# A preliminary report on dental implant condition among dependent elderly based on the survey among Japanese dental practitioners

**DOI:** 10.1186/s40729-018-0125-7

**Published:** 2018-05-08

**Authors:** Yuji Sato, Shigeto Koyama, Chikahiro Ohkubo, Shin Ogura, Ryutaro Kamijo, Soh Sato, Jun Aida, Yuichi Izumi, Mihoko Atsumi, Akio Isobe, Shunsuke Baba, Noriharu Ikumi, Fumihiko Watanabe

**Affiliations:** 1 Department of Geriatric Dentistry, Showa University School of Dentistry, 2-1-1, Kitasenzoku, Ohta-ku, Tokyo, 145-8515 Japan; 2 Maxillofacial Prosthetics Clinic, Tohoku University Hospital, 1-1, Seiryomachi Aoba-ku, Sendai-shi, Miyagi 980-8574 Japan; 3 Department of Removable Prosthodontics, Tsurumi University School of Dental Medicine, 2-1-3, Tsurumi, Tsurumi-ku, Yokohama-shi, Kanagawa 230-8501 Japan; 4 Division of Oral Implant, The Nippon Dental University Hospital Tokyo, 2-3-16, Fujimi, Chiyoda-ku, Tokyo, 102-8158 Japan; 5 Department of Biochemistry, Showa University School of Dentistry, 1-5-8, Hatanodai, Shinagawa-ku, Tokyo, 142-8555 Japan; 6 Department of Periodontology, The Nippon Dental University School of Life Dentistry at Niigata, 1-8, Hamauracho, Chuo-ku, Niigata-Shi, Niigata, 951-8580 Japan; 7 Department of International and Community Oral Health, Tohoku University Graduate School of Dentistry, 4-1, Seiryomachi Aoba-ku, Sendai-shi, Miyagi 980-8575 Japan; 8 Department of Periodontology, Tokyo Medical and Dental University Graduate School of Medical and Dental Sciences, 1-5-45, Yushima, Bunkyo-ku, Tokyo, 113-8510 Japan; 9 Department of Oral Interdisciplinary Medicine, Kanagawa Dental University Graduate School of Dentistry, 82, Inaokacho, Yokosuka-shi, Kanagawa 238-8580 Japan; 10 Department of Oral Implantology, Osaka Dental University, 1-5-17, Otemae, Chuo-ku, Osaka-shi, Osaka, 540-0008 Japan; 11 Medical Corporation Ishikura Dental Clinic, 457-3, Iizukamachi, Takasaki, Gunma 370-0069 Japan; 12 Department of Crown & Bridge Prosthodontics, The Nippon Dental University School of Life Dentistry at Niigata, 1-8, Hamauracho, Chuo-ku, Niigata-Shi, Niigata, 951-8580 Japan

**Keywords:** Older adults, Implant, Home-visit dental care, Implant card

## Abstract

**Background:**

The objective of this study was to ascertain the situation relevant to implants, the status of oral self-care, the status of aftercare provided by the dentist who placed the implant, and the usage status of the implant card, in homebound or institutionalized older adults who are receiving home-visit dental care due to the inability to visit a dental clinic on their own.

**Methods:**

A survey questionnaire was sent by post mail to 2339 people who are representative members or dental specialists belonging to any of the following three academic societies: Japanese Society of Oral Implantology, Japanese Society of Gerodontology, and Japan Prosthodontic Society. The survey questions asked were about provision/no provision of implant treatment, provision/no provision of home-visit dental care, the situation of patients after implant treatment, the situation of implants in the context of home-visit dental care, and the usage status and recognition of the implant card.

**Results:**

No less than 30% of the dentists had patients who were admitted to the hospital or became homebound after receiving implant treatment at their clinic. Twenty-two percent of the dentists had been consulted about the implants. Dentists who continued to provide post-operative implant care through home-visit dental care accounted for approximately 80%. On the other hand, however, 40% of the dentists did not know the post-implantation status of their implant patients. Of the patients receiving home-visit dental care, approximately 3% had implants (identified mainly by visual inspection). It was found that more than 50% of the dentists offering implant treatment did not use the implant card, and even in cases where it was used, most of the cards were not in the standardized format.

**Conclusions:**

Within the limitation of low response rate to the questionnaire in this preliminary study, we concluded that many of practitioners including specialists indicated the need of universal record of implant for dependent elderly cares.

## Review

### Background

In September 2016, Japan’s graying population reached a level where 27.3% (34.61 million) of the total population was 65 years or older, as announced by the Statistics Bureau of the Ministry of Internal Affairs and Communications [1]. Of the older adults, over 6 million people or approximately 20% require long-term care [2]. These elderly people have difficulty attending clinics, which easily results in worsening of intraoral conditions. Meanwhile, the prevalence of dental implants is rising; the *Survey of Dental Diseases* for fiscal year 2011 [3] reported that 3% of older adults have implants. However, the 4253 survey respondents included only 1510 older adults, and older people who were institutionalized in long-term care homes were excluded from the research. Therefore, the results of the survey have not yet revealed a comprehensive picture of the status of dental implants in the elderly population receiving long-term care.

Treatment with implants in itself has been successfully performed even in older adults [4] and people with disabilities [5] as long as the patients are appropriately managed. It is likely that older adults who are receiving long-term care and unable to travel to the dentist’s office have difficulty continuing to perform oral self-care and receive professional oral care due to the complexity of the form of prosthesis and the problem of the implant placement [6, 7]. Accordingly, in a study of three case reports, Visser et al. indicated the importance of considering the following aspects: “Is the patient supported by a well-functioning oral (self) care assisting network?” and “Is it possible for the patient to regular see an oral health care professional and is oral health care easily accessible in cases of an emergency?” [8]. In addition, for the fixed implant prosthetic devices which have been selected for the patient who is getting old, the original prosthetic devices have to be removed and may be changed to removable prosthetic devices or submerged implants when self-care becomes difficult or when having trouble with the prosthetic device [7]. However, if the manufacturers or type of the implants in patients are not sure, it might be difficult to change the design of the prosthesis.

Therefore, a survey was carried out to investigate the rate of having received implant treatment in receiving long-term care or home-visit dental care patients and the actual status of oral self-care, as well as the actual status of post-operative care by the dentist who placed the implants, in homebound or institutionalized older adults who are receiving home-visit dental care due to the inability to visit a dental clinic on their own. Additionally, usage status and recognition of the implant card (it refers to the card which described the record of implant placed in the patient, such as the implant manufacturer, implant type, length, diameter), which contains information on the implanted implant and may contribute to continuing post-operative implant care, were surveyed.

### Methods

The survey was conducted during 3 months from August to October 2015 by non-anonymous questionnaire (four pages on A4 paper) including questions developed by the authors of the present study (Table 1). The survey sheets were sent and collected by post mail.

**Table 1:** Survey questions

1. Do you offer implant treatment?
2. Do you give a “card/pocket notebook” to patients for whom implant treatment has been completed?
3. Among the patients who received implant treatment at your clinic, are there any patients who were admitted to the hospital or became bedridden at home?
4. Have you been consulted by your implant patients or their families about oral health management when the patients were admitted to the hospital or became bedridden?
5. If you are informed by one of your implant patients that s/he cannot visit your clinic due to becoming bedridden, how do you address this?
6. Please provide the number of institutions and patients by the category of institutions you visit for home-visit dental care.
Number of institutions
Total number of patients who receive your home-visit dental care
Of the above patients, the total number of patients who are unable to perform oral self-care
Total number of patients who have implants among those who receive your home-visit dental care
Of the above patients who have implants, the total number of patients who are unable to perform oral self-care
7. How do you identify the presence of implants in patients receiving your home-visit dental care?
8. Would it be helpful if institutionalized or homebound older adults have an implant card/pocket notebook (something like the Prescription Pocket Notebook) or treatment history/information?

**Fig. 1:**
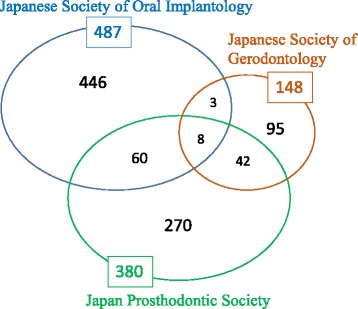
Breakdown of respondents. The retrieval rate was approximately 40% in each of the three societies

The target population of the survey was 2339 representative members or dental specialists belonging to any of the following three organizations: Japanese Society of Oral Implantology and Japanese Society of Gerodontology as academic societies relevant to implants and home-visit dental care, respectively, and Japan Prosthodontic Society as an academic society related to both fields. A total of 924 people responded to the questionnaire (retrieval rate 40%). Figure 1 gives a breakdown of the societies to which the respondents belonged.

The present study examined the following four aspects related to implant patients and home-visit dental care.

1. The situation of hospitalization/in-home convalescence as well as consultation about post-operative implant care sought by patients’ families and how dentists address this.
2. Proportion of individuals who have implants, the situation of oral self-care, and the dentists who provided implant treatment, among patients receiving home-visit dental care.
3. Methods to identify the presence of implants in patients receiving home-visit dental care.
4. Usage status of the implant card retained by the above patients (“implant card” refers to the card which described the record of implant placed in the patient, such as the implant manufacturer, implant type, length, diameter. This questionnaire does not require a publisher, such as manufacturers and a society. If the respondent replies that the implant card had been used, he/she had written the publisher.).

Analyses of the relationship between necessary variables were performed by the *χ*^2^ test.

The present study was carried out with the approval of the Ethics Committee of the Japanese Society of Oral Implantology (Number 2015-1).

### Results

**Fig. 2:**
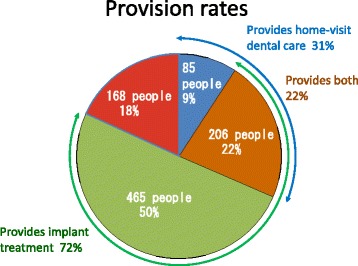
Three implants were embedded in an artificial mandible

Of the 924 dentists participating in the survey, 465 respondents (50%) offer implant treatment only and 85 respondents (9%) provide home-visit dental care only. Two hundred and six respondents (22%) provide both implant treatment and home-visit dental care (Fig. 2). The number of dentists who provide home-visit dental care was significantly lower among those who offer implant treatment (*p* < 0.01).

1. The situation of hospitalization/in-home convalescence as well as consultation about post-operative implant care sought by patients’ families and how dentists address this.

Table 2 presents the percentage of the dentists’ answers as to the extent of the presence of patients who received implant treatment on an outpatient basis but subsequently were admitted to the hospital or became homebound. Thirty percent of the dentists had implant patients who were admitted to the hospital or became homebound, and 27% of the dentists had no such patients, while 41% of the dentists had no knowledge about this.

**Table 2:** Among the patients who received implant treatment at your clinic, are there any patients who were admitted to the hospital or became bedridden at home?

	The number of respondents
Yes, there is	204 (30%)
No, there isn’t	182 (27%)
I don’t know	278 (41%)
No answer	7 (1%)

Table 3 shows the proportion of dentists who had been consulted by implant patients or their families about oral health management when the patients were admitted to the hospital or became bedridden. Only 22% of the dentists had been consulted by the families of implant patients who were admitted to the hospital or became homebound. Seventy-six percent of the families of these patients had not sought counseling. The contents of the consultation were mainly about the “cleaning method/management method.”

**Table 3:** Have you been consulted by your implant patients or their families about oral health management when the patients were admitted to the hospital or became bedridden?

	The number of respondents
Yes, I have	150 (22%)
No, have not	513 (76%)
No answer	8 (1%)

Table 4 illustrates the percentage of answers to the question, “If you are informed by any of your implant patients that they cannot visit your clinic because they have become bedridden, how do you address this?”. Approximately 80% of the dentists answered that they would provide the post-operative care themselves or they would ask another dentist to provide the care instead of them. However, dentists who gave answers that would not lead to the provision of home-visit dental care accounted for 20%.

2. Proportion of individuals who have implants, the situation of oral self-care, and the dentists who provided implant treatment, among patients receiving home-visit dental care.

**Table 4:** If you are informed by one of your implant patients that s/he cannot visit your clinic due to becoming bedridden, how do you address this?

	The number of respondents
I’ll do nothing	34 (5%)
I’ll ask another dentist to provide the patient with home-visit dental care	217 (32%)
I’ll advice the patient to perform oral self-care only	111 (17%)
I’ll continue to provide the patient with post-operative care through home-visit dental care	326 (49%)
Others	59 (9%)
No answer	26 (4%)

Table 5 shows the dentists’ answers to the question, “Have you actually seen provided implants for patients while providing home-visit dental care over the past 12 months?” Two hundred and ninety-one dentists had provided dental care at 4569 institutions and had seen a total of 12,356 patients, of whom 3% had implants. Of the implant patients, those who had their implants placed at the dentist’s dental clinic accounted for only approximately one-third (31%). Additionally, of all the patients, as many as 8795 patients were unable to perform oral self-care on their own. Among the patients with implants (360), the proportion of those who were unable to perform self-care was 56% (200), which was significantly lower (*p* < 0.01) compared to 77% for the patients who had no implants.

3. Methods to identify the presence of implants in patients receiving home-visit dental care.

**Table 5:** Number, implants (whether placed by the visiting dentist), and oral self-care of patients receiving home-visit dental care seen by dentists who provided home-visit dental care over the past 12 months (291 dentists, 4,569 institutions)

	Total number of patients	Patients with implants	Patients with implants placed by the visiting dentist
Total number of patients	12,356	360 (3%)	112 (31%)
Patients who cannot perform oral self-care	8,795 (71%)	200 (56%)	

Table 6 shows the methods used by the dentists who provide home-visit dental care to identify the presence of implants in patients. The most common method was “visual inspection.” On the other hand, use of radiography was 17%.

4. Usage status of the implant card retained by patients.

**Table 6:** Methods to identify the presence of implants in patients receiving home-visit dental care

	The number of respondents
Visual inspection	153 (53%)
Radiography	49 (17%)
Information provided by patients or their families	88 (30%)
Ask patients’ dentists	10 (3%)
Implant card	5 (2%)
Dental records of implant surgeries that I performed	46 (16%)
Others	11 (4%)
No answer	50 (17%)

The implant card was evaluated as effective by the majority of dentists who provide home-visit dental care, regardless of whether or not they had seen patients with implants (Table 7). However, of the 671 dentists who offer implant treatment, those who were using the implant card (or pocket notebook) accounted for less than 50% and the dentists who were utilizing the standardized card (developed by the Japanese Society of Oral Implantology) or pocket notebook (developed by the Japanese Academy of Maxillofacial Implants) accounted for only 10% (Table 8).

**Table 7:** Recognition on the effectiveness of implant card

	Seen patients with implants (person)	Never seen patients with implants (person)
Very effective	44	67
Effective	27	44
A little effective	14	7
Useless	0	2
No answer	13	73

**Table 8:** Survey of dentists who offer implant treatment (671 dentists). “Do you use the implant card?”

	The number of respondents
The card developed by the Japanese Society of Oral Implantology	54 (8%)
The pocket notebook developed by the Japanese Academy of Maxillofacial Implants	11 (2%)
Implant card developed by manufacturers	133 (20%)
Unique implant card made by the dentist	121 (18%)
No supply	360 (54%)

Regardless of whether or not the dentist provides home-visit dental care

### Discussion

Questionnaire survey by post mail had been said to be low retrieval rate, but it was considered to be suitable for information response of many clinics and hospitals [9]. In this research, a questionnaire survey was conducted for representative members or dental specialists of three academic societies who are likely to understand the significance of the research and are thought to be engaged in implant treatment or home-visit dental care. Therefore, the questionnaire retrieval rate of this survey was close to other similar questionnaire survey [10], and it might have little confusion of question contents of the questionnaire. Further considerations are to further improve the response rate of the questionnaire and the actual situation of visiting dental practice other than this subject.

The present study found that no less than 30% of the dentists had patients who were admitted to the hospital or became bedridden at home after receiving implant treatment at their clinic and that 22% of the dentists had been consulted about the implants. Dentists who had provided continued post-operative implant care through home-visit dental care accounted for approximately 80%, whereas 40% of the dentists did not know the post-implantation status of their implant patients. Moreover, because the dentists who answered “I have no patients that were admitted to the hospital or became bedridden at home” (27%) are likely to include those who were not aware of such patients, it can be inferred that a larger number of dentists do not know the status of their implant patients after placement of the implants, which suggests the need to facilitate an understanding of the post-implantation status of patients.

Approximately 3% of the patients seen in home-visit dental care had implants. Lantto et al. reported a lower proportion of having implants among older adults receiving long-term care compared to healthy controls [11]. Meanwhile, the rate of having implants among older adults that was calculated based on the results of the *Survey of Dental Diseases* is approximately 3%, which is largely consistent with the results of the present study among the older adults receiving long-term care. However, as the results of the present study and the *Survey of Dental Diseases* were mostly examined by visual inspection, it could not deny the possibility that have failed to detect potentially more implants, it is necessary to investigate the actual situation in the future. Fifty-six percent of the patients with implants were unable to perform oral self-care, which is lower compared to 77% in patients without implants. The outcome may be attributable to a high level of interest in the oral condition seen in patients with implants as well as the difference in the general condition such as age, cognition function, and cerebrovascular disease between the two groups, which is a subject for future research. However, the percentage, 56%, in itself is a high level as the proportion of patients incapable of oral self-care, and hence, it is important to provide them with professional care/management despite the issue of manpower [12, 13]. Moreover, some case, it is difficult for elderly patients or carers to clean their implant-supported prosthesis [8], so it is important to elucidate how the position or number of implants influences the complexity of care. Therefore, it might be necessary to promote a unified and standardized implant card describing necessary and sufficient implant information.

In addition, the number of patients with implants placed at the dentist’s own clinic accounted for only one third, suggesting that the remaining two thirds of the patients had the implants placed at other dental clinics. Ideally, this implies a great need for the standardization on size and shape of screw or driver used for implants, which would likely be useful to ensure continued post-operative implant care. It will be necessary to encourage implant manufacturers to do so through academic societies and dental associations. However, since basic research on mechanical performance is also necessary, it is difficult to promptly promote it, so it seems realistically to promote standardized implant cards. Even the dentists who do not offer implant treatment clearly recognized the need for implant cards, as Visser et al. [8] suggested the necessity for an “implant passport” [7]. In actuality, however, more than 50% of the dentists offering implant treatment do not use the implant card and, even in cases where it is used, most of the cards are not standardized ones. Thus, we strongly hope that a systematized implant card will gain widespread use.

It is necessary in the future to elucidate implant-related problems arising in home-visit dental care and how they are actually addressed.

## Conclusion

Within the limitation of low response rate to the questionnaire in this preliminary study, we concluded that many of practitioners including specialists indicated the need of universal record of implant for dependent elderly cares.
